# High gain and high efficiency soft switching quadratic boost converter for renewable energy applications

**DOI:** 10.1038/s41598-025-20207-2

**Published:** 2025-10-16

**Authors:** Farag Elghabsi, Mohd Rodhi Sahid, Razman Ayop, Ahmed Alanssari, Mohammad Al Takrouri, Mahmood Swadi, Masoud A. Sahhouk, Hasmat Malik, Vinay Kumar Jadoun

**Affiliations:** 1 Department of Electrical Power Engineering, Faculty of Electrical Engineering, Universiti Teknologi Malaysia (UTM), Skudai 81310, Johor Malaysia; 2 Department of Electrical Engineering, College of Engineering, University of Baghdad, Baghdad, 10001 Iraq; 3 Department of Electrical & Electronics Engineering, Manipal Institute of Technology Manipal Academy of Higher Education, Manipal, India; 4 Department of Electrical Engineering, Graphic Era (Deemed to be University), Dehradun 248002, India

**Keywords:** Quadratic boost converter, High voltage gain, Steady-state analysis, Soft switching, Electrical and electronic engineering, Renewable energy

## Abstract

Quadratic boost converter (QBC) is crucial in immediate technologies, including renewable energy, electric vehicles (EV), DC microgrids and EV charging stations, where efficient and dependable power conversion is essential. This article introduces a high-gain, high-efficiency QBC operating with soft-switching capabilities explicitly tailored for renewable energy sources that can be used in charging stations for EVs. The design achieves high voltage gain (VGN) by incorporating a coupled inductor (CIN) with a restricted duty cycle, which minimizes the need for extreme duty cycle adjustments that often impact efficiency and cause component stress. The leakage inductances of the CIN’s enable zero voltage switching (ZVS) for the power switches at turn-on and ZVS turn-on and zero current switching (ZCS) turn-off for the diodes. This approach mitigates the switches’ losses and enhances the efficiency. Furthermore, an active clamp circuit is employed, allowing the converter to operate with reduced voltage stress across semiconductor components, thus improving their durability and reliability. The converter operated in continuous conduction mode (CCM) is extensively analysed to assess its performance across various operating conditions. This converter compares VGN, efficiency, and stress on components with several recent QBCs for a comprehensive performance assessment. The converter’s 250 W hardware prototype has also been built and tested, demonstrating its practical suitability and effectiveness for high-demand renewable energy applications. Experimental findings affirm the converter’s high efficiency and reliable performance in real-world situation.

## Introduction

Conventional electricity generation has many drawbacks, including extensive land use for power generation, transmission, and distribution, harmful waste from nuclear power plants, high greenhouse gas emissions from fossil power plants, and excessive water use in thermal power plants. Renewable energy sources (RESs) are suggested as the future solution to sustainable energy. RESs produce clean energy due to their zero carbon footprint, making them environmentally friendly^1^. However, these sources’ availability is stochastic, affecting their effective utilization^2–4^. In addition, the power produced from these sources is mainly at a minimal voltage level^5^. To maximize the advantages of these sources, power electronics converters are essential for generating usable power that satisfies user requirements. Consequently, power converters, particularly DC converters, are necessary for stabilizing the power of these sources^3^. Several configurations of converters are reported in literature. These converters are simple in structure and control and have a low component count, translating to low-power processing stages and low-cost^5^. Despite these benefits, these converters have a limited voltage conversion ratio. Hence, (VGN) DC converters are desirable in several applications. To enhance (VCR) with a standard boost converter, the cycle of the controlled switch should be higher^6,7^. Thus, increasing the duty cycle boost the conduction loss and reduce the efficiency. Furthermore, larger duty cycle limits the converter switching frequency because of the short time needed to power the switch. Similarly, electromagnetic interference (EMI) related issues are produced due to severe reverse recovery (Rr) loss of the diodes.

In general, high voltage boosting with a single converter imposes a constraint on the efficiency due to limitations on duty cycle control and an increase in current ripple^6,8,9^. Furthermore, high switching loss is another setback of conventional converter. Various DC-DC converters without extreme duty cycles are identified in the literature to address the abovementioned limitations. In^10–13^, high frequency transformers are used to increase the VGN. These converters use transformer for voltage scale up and ensure safety by separating the low and high-voltage. The high VGN could be obtained by boosting the transformer $\:\left(n\right)$. Consequently, the transformer is the bulkiest part of a converter; thus, increasing the $\:\left(n\right)$ expands the size, weight, and leakage reactance, which lowers the efficiency. Combining multiple converters in series/parallel or series-parallel^14–16^ is another way of increasing the output power. On the contrary, this approach increases the number of component counts, system cost, and reduction in efficiency due to loss attributed to different individual components. The cascaded connection of two boost converter stages is another method of increasing a converter voltage gain with moderately high efficiency without extending the duty cycle, as reported in^17–19^. Unfortunately, this concept complicates the design of the compensator with the involvement of multiple active switches. Furthermore, cascaded techniques require increasing components and significantly reducing efficiency^20^.

Non-isolated converters using coupled inductors (CIN) to increase VCR by increasing the $\:\left(n\right)$ of the CIN are reported in^20–23^. In^21^, the converter uses a CIN to maintain a continuous output current; however, the leakage energy of the CIN induces a high (VSS) on the switches. The converter reported in^23^ utilizes two coupled inductors to increase the VGN and a regenerative snubber to imbibe the stray inductance energy. However, the snubber in the circuit increases converter complexity. Furthermore, the topology reported in^33,34^ integrates a voltage multiplier cell (VMC) to enhance the VGN, however, these converters uses three magnetic cores which reduce the power density. The topology proposed in^26^ achieves significant increase in the converter gain with the help of a three winding CINs. In addition, the $\:{L}_{LK\:}$ of the CIN is used to acquire ZVS for the power switch. However, the three windings CIN increase the converter weight and lower the power density. Other methods of achieving high VGN are based on voltage multiplier cells and an interleave structure. In^24^ a converter with a switch capacitor multiplier cell is reported. The converter used capacitor multiplier cells to boost static VCR without raising the duty cycle. However, increasing the gain comes with the price of adding more switches and capacitors, thus increasing the converter cost, volume, and conduction loss. Similarly, the indiscriminate use of capacitor cells at the converter’s input makes the input current exhibit a pulsating behaviour^28^. Although, these drawbacks can be compensated by using a diode switch capacitor at the load side, at the expense of higher converter volume and cost^29^. Consequently, the voltage level is restricted by high current stress and high switching loss across the switch. In^27^ interleaving converters combining switch capacitor cells with coupled inductors are reported to boost the converter gain, however, the topology has a low power density and high component count.

A non-isolated QBC with a VGN as a quadratic function of a conventional boost converter is studied and reported in^24–26,31–35^. The QBC-based CUK topology proposed in^31^ has a constantly operating input current, few components, and a straightforward control structure. However, the power switch is exposed to a VSS comparable to the output voltage. Conversely, the topology proposed in^32^ uses a single active switch with minimal isolated capacitor voltage stress and a quadratic voltage transfer ratio comparable to the standard converter. Conversely, the VCR of the converter is unsuitable to use in VGN applications. Furthermore, the control switch operates under hard switching condition, increasing the loss and degrading efficiency. The topology proposed in^35^ increases the static gain with fewer components using a three-level QBC. In consequence, voltage imbalance across the output capacitors affects the circuit operation. Although the closed-loop controller can mitigate this, the circuit complexity is increased. Additionally, each power switch is on-off with hard switching condition. Similarly, the reconfigured topology reported in^33^ obtained a VGN as a quadratic coefficient of the $\:D$. Consequently, all the switches are on-off with hard switching conditions and this increases the converter loss. In addition, the VSS on the $\:{D}_{o}$ and the switch are both equal to the $\:{V}_{O}$.

Despite the remarkable advantages of non-isolated QBC converters, the converters face problems such as high switching loss, high VSS across power switches, high component count, and output currents and non-pulsating input. This article suggested a high VGN converter in a QBC structure. The suggested converter uses a voltage multiplier structure and a couple of inductors, resulting in the following contributions:

1. Compared to the existing literature, the suggested converter produces a high gain ratio QBC with coupled inductors at a low $\:D$.
2. The proposed converter exhibits a low VSS across the power switches which extends the lifespan of semiconductor devices.
3. The suggested topology operates under ZVS mode, reducing the switching loss and improving efficiency.
4. The converter possesses a common ground between output and input, which simplifies the design of controller.

This is how the article is structured: Part II provides a proposed approach. Part III models the proposed converter. Part IV explains the selection criterion. Part V Determination of stress across the components and VI presents the simulation and experimental results, while Part VII provides the particular application and suitability. Part VIII provides the comparative analysis. Finally, IX provides the conclusion.

## Proposed approach

**Fig. 1:**
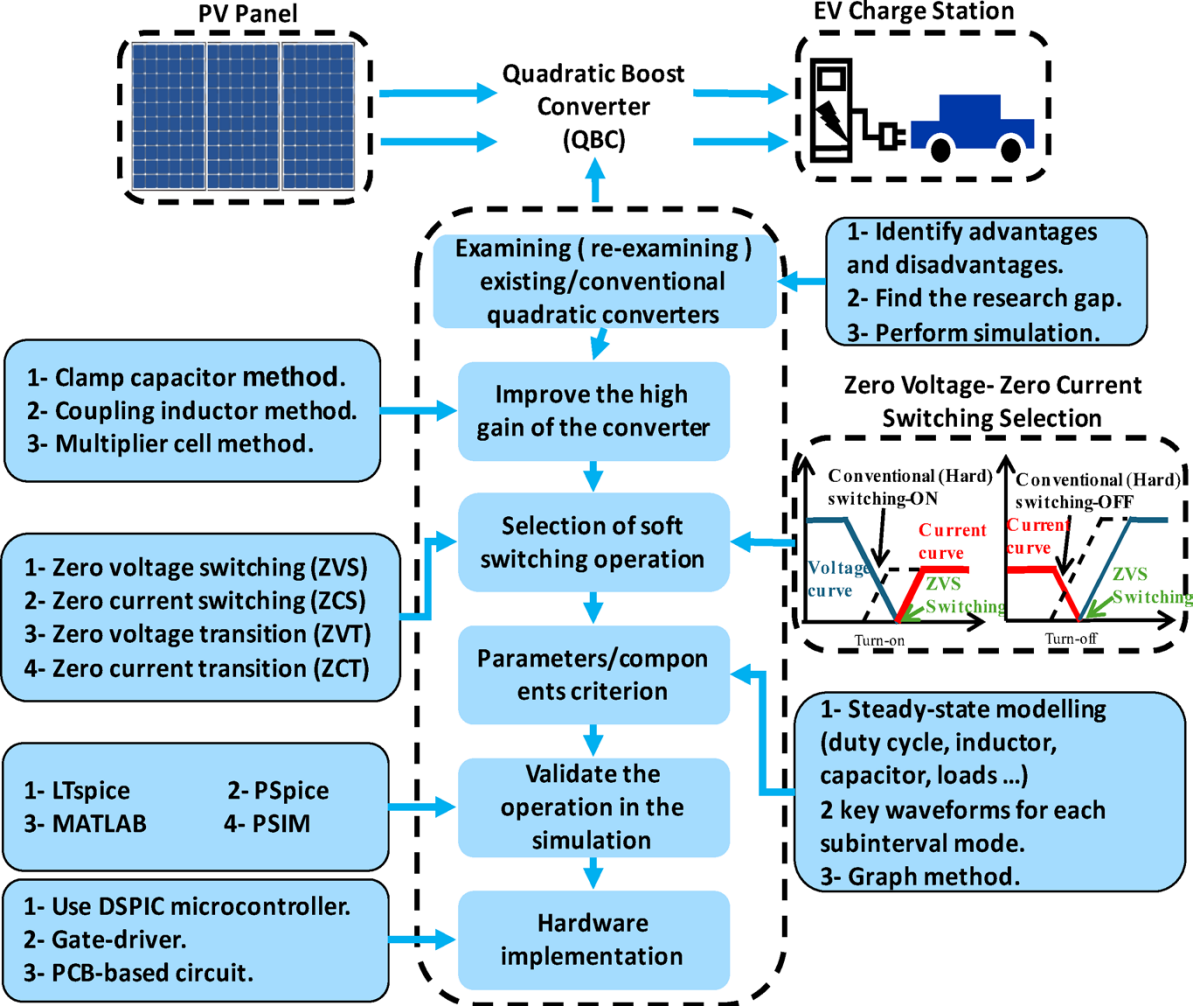
Proposed design approach.

This flowchart outlines the design and development process for a QBC used in applications like photovoltaic (PV) systems and (EV) charging stations. It begins with examining existing QBC designs to identify research gaps, followed by methods to improve high gain, such as clamp capacitors, coupling inductors, or multiplier cell methods. (ZVS), (ZCS), and (ZVT/ZCT) are then considered for efficiency. All this is shown in Fig. 1. Critical design criteria including component parameter selection, steady-state modelling, and waveform analysis. The design is validated through simulations using PSpice software and subsequently implemented in hardware. This comprehensive process ensures high efficiency, reduced switching losses, and reliable operation for power electronics applications.

### Description of the proposed converter

Classical QBC operates at a limited $\:D\:$to mitigate high current ripple, (Rr) problems of the diode, and high switching loss. However, the converter is impacted by some drawbacks, including hard switching of power switches, excessive voltage stress across devices, and limited voltage conversion ratio, which limits its use in applications requiring high voltage.

**Fig. 2:**
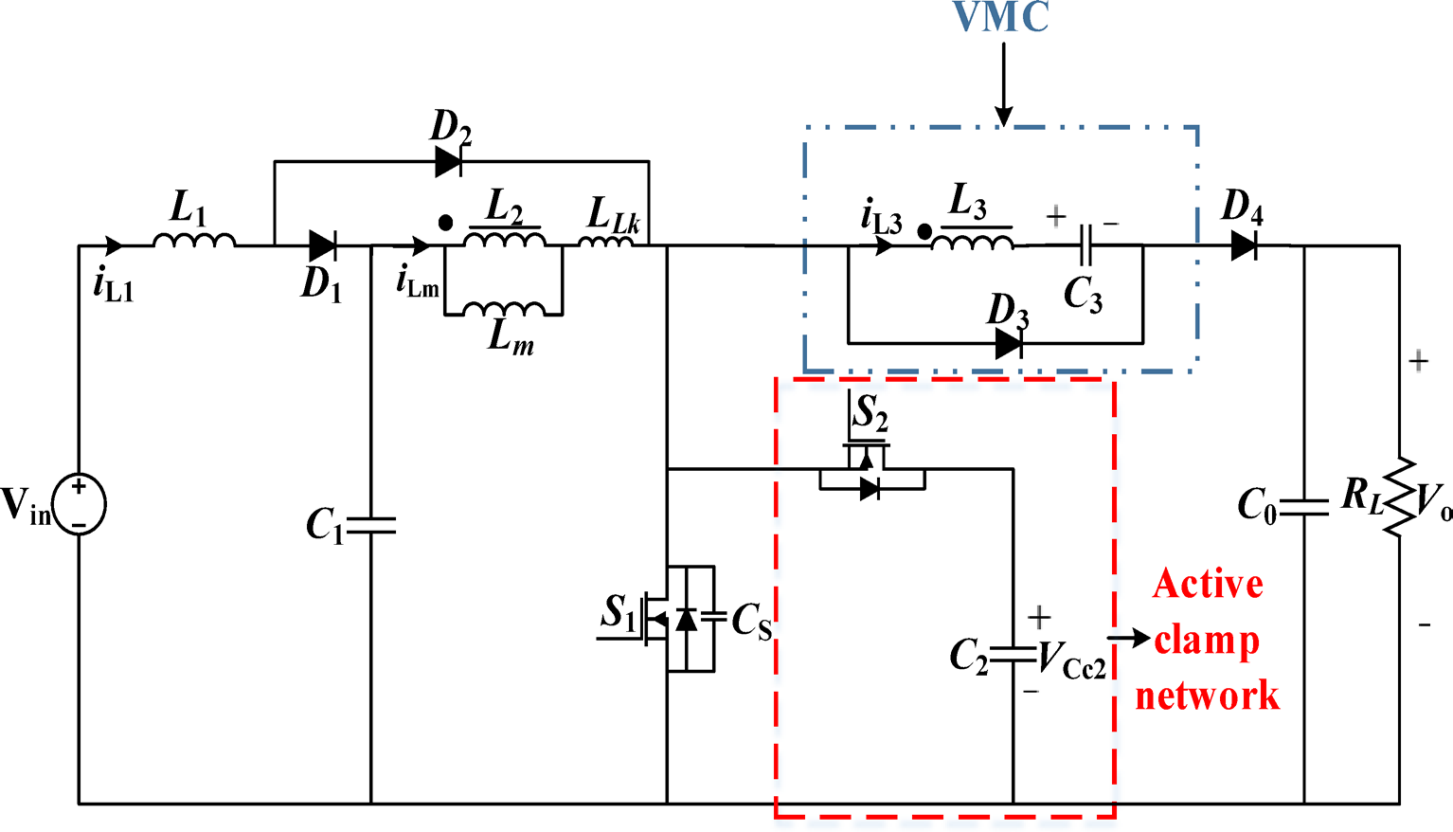
Proposed high voltage gain QBC converter.

A high VGN non-isolated soft switching QBC is proposed to address these drawbacks. The converter topology employs an active clamp network and VMC to increase the VCR and achieve ZVS for $\:{S}_{1,\:}\:,\:{S}_{2}$ and ZCS for the diodes, respectively. Furthermore, the switches are subjected to low VSS because of the active clamp circuit. The proposed QBC consists of an input inductor $\:{L}_{1},$ and coupled inductors modelled as transformers comprising primary and secondary turns ($\:{L}_{2}\:$and$\:\:{L}_{3}$), ($\:{D}_{1},\:{D}_{2},\:{D}_{3},\:$and$\:{\:D}_{4}$) and ($\:{C}_{1},\:{C}_{2},{C}_{3},\:$and $\:{C}_{0}$) respectively. The presence of $\:{L}_{LK}\:$in the switches and diodes naturally provide ZVS along the current flow. The suggested converter’s diagram is shown in Fig. 2.

## Modelling the suggeeted converter

The following presumptions guide the study of the psuggested converter:

1. The value of $\:{C}_{1}$, $\:{C}_{2},$ $\:{C}_{3}$ and $\:{C}_{0}$ are large enough to generate a steady voltage.
2. All semiconductor devices are ideal.

**Fig. 3:**
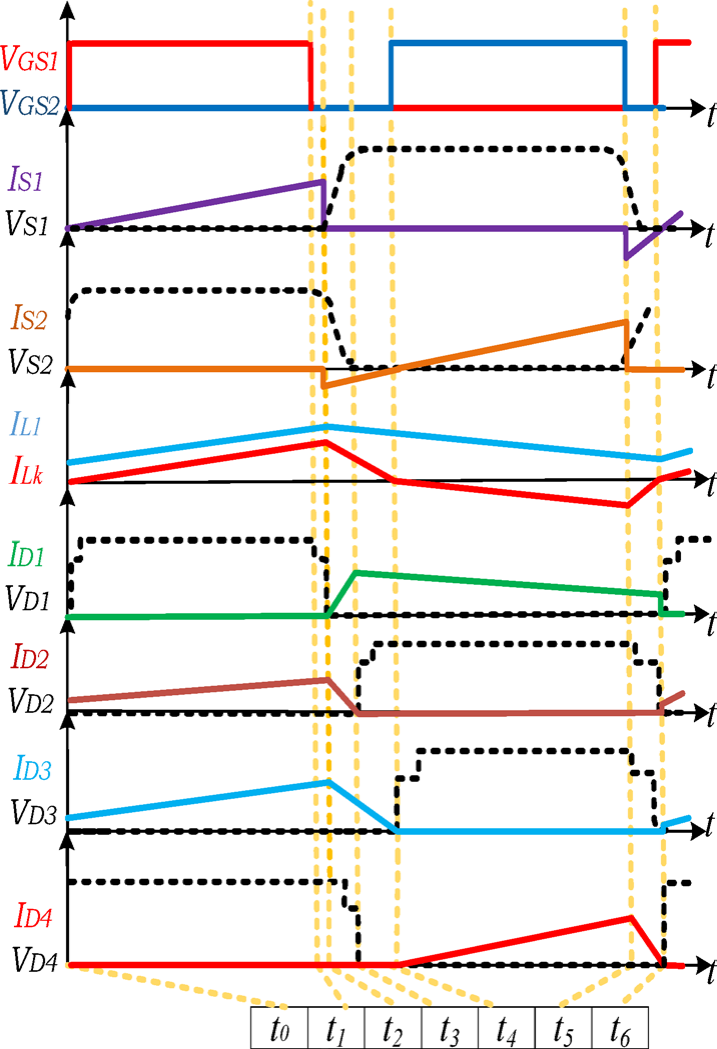
Key waveforms of the proposed converter.

**Fig. 4:**
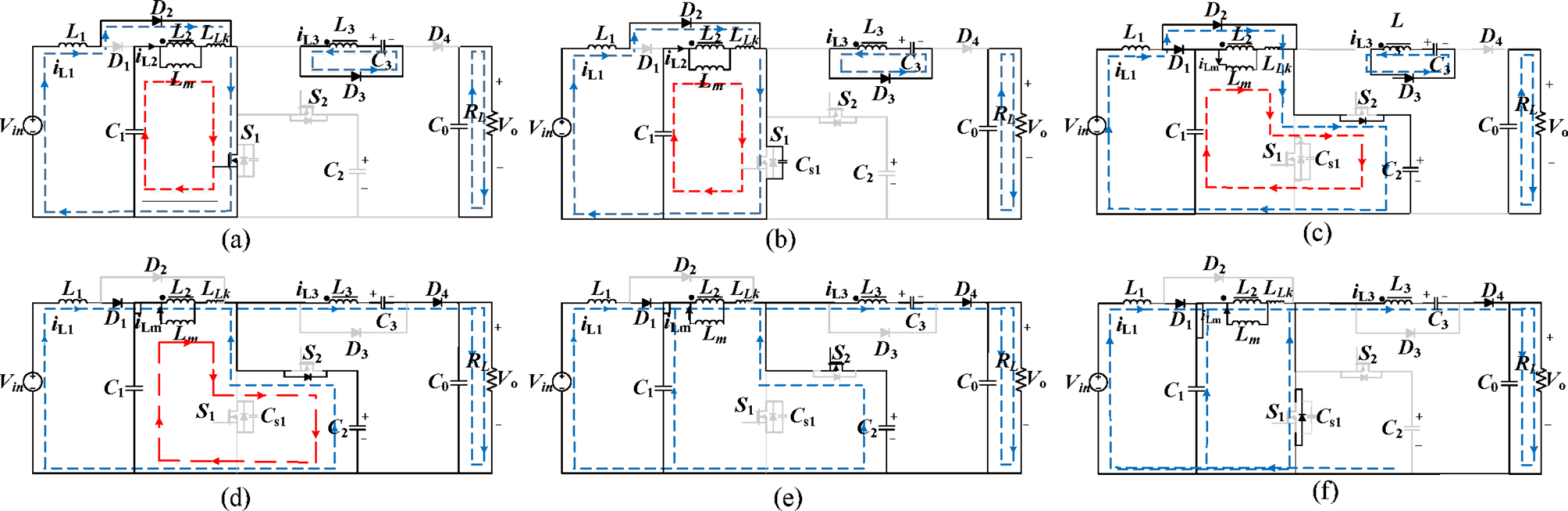
Equivalent diagram of the operating modes (a) Mode 1, (b) Mode 2, (c) Mode 3, (d) Mode 4, (e) Mode 5, (f) Mode 6.

Figure 3 shows the key waveforms of the proposed converter that display each of the operating modes. Similarly, Fig. 4 shows the equivalent circuit of the various operating modes.

Mode 1 ($\:{t}_{0}<t<{t}_{1})$: in Fig. 4 (a), prior to this mode, the current crosses the $\:{i}_{Lk}$ charged the body capacitor of the $\:{S}_{1}$ and the $\:{S}_{1}$ is tuned on with ZVS.$\:{L}_{m}$ and $\:{L}_{1}$ are linearly charged through sources $\:{V}_{C1}\:$and$\:{V}_{in.}$ The following equations describe the inductor current:

1 $$\:{i}_{L1}\left(t\right)={i}_{L1}\left({t}_{0}\right)+{V}_{in\:}/{L}_{1}(t-{t}_{0})$$

2 $$\:{i}_{Lm}\left(t\right)={i}_{Lm}\left({t}_{0}\right)+{V}_{C1}\:/{L}_{m}\left(t-{t}_{0}\right)$$

3 $$\:{i}_{Lk}\left(t\right)={i}_{Lk}\left({t}_{0}\right)+{V}_{C1}-\:\frac{{V}_{C3}}{n}\:/{L}_{Lk}\left(t-{t}_{0}\right)$$

$\:{C}_{0}$ is discharged through the load resistance. Mode 1 ends when $\:{S}_{1}$ turn off at $\:{t}_{1}$. At time $\:{t}_{1}$ the inductors currents is stated as.

4 $$\:{i}_{L1}\left({t}_{1}\right)={i}_{L1}\left({t}_{0}\right)+{V}_{in}/{L}_{1}DT$$

5 $$\:{i}_{Lm}\left({t}_{1}\right)={i}_{Lm}\left({t}_{0}\right)+\frac{{V}_{C1}}{{L}_{m}}DT$$

6 $$\:{i}_{Lk}\left({t}_{1}\right)={i}_{Lk}\left({t}_{0}\right)+{V}_{C1}-\:\frac{{V}_{C3}}{n}/{L}_{Lk}DT$$

where $\:DT$ is the $\:D$ on time of and switching phase for the switch $\:{S}_{1.}$

Mode 2 ($\:{t}_{1}<t<{t}_{2})$: In Fig. 4 (b), it starts at $\:{t}_{1}$ when $\:{S}_{1}$ is off and $\:{i}_{L1}$, $\:{i}_{Lm}$ and $\:{i}_{Lk}$ increase linearly. The $\:{i}_{Lk}$ charges the body of $\:{C}_{S1}$ to approximately $\:{V}_{C1}$. $\:{C}_{1}$ and $\:{C}_{S1}$ starts to resonate with the magnetizing inductor $\:{L}_{m}$, this mode ends at $\:{t}_{2}$ with $\:{i}_{ds1}=0.$ The current equation for the magnetizing inductor and the voltage in $\:{C}_{s1}$ are provided by:

7 $$\:{i}_{Lk}\left(t\right)=1/{Z}_{o}\left[\left({V}_{in}-\frac{{V}_{C3}}{n}\right)+\frac{{C}_{S1}}{{C}_{1}+{C}_{S1}}.{V}_{Cs1}\right]\:\text{sin}{{\upomega\:}}_{0}\left(t-{t}_{1}\right)+{i}_{{L}_{k}}\left({t}_{1}\right)\text{cos}{{\upomega\:}}_{0}\left(t-{t}_{1}\right)$$

where$\:\:{Z}_{o}$ given as:

$$\:{Z}_{o}=\sqrt{\frac{{L}_{Lk}}{{C}_{S1}}}\:{{\upomega\:}}_{0}=\frac{1}{\sqrt{{C}_{S1}.{L}_{LK}}}$$

Mode 3 ($\:{t}_{2}<t<{t}_{3})$: In Fig. 4 (c) starts at $\:{t}_{2}$ when diode $\:{D}_{1}$and $\:{D}_{2}$ are forward bias and inductor current$\:{i}_{Lk}$ linearly decreasing towards zero through the diode of the $\:{S}_{2}$. This will ensure ZVS turns on $\:{S}_{2}$ due to zero drain-to-source voltage $\:\left({v}_{ds2}=0\right)$ of switch $\:{S}_{2}$. Furthermore, due to the conduction of $\:{D}_{1}$ and $\:{D}_{2}$, the current passing throughout the $\:{L}_{Lk}$, $\:{i}_{Lk}$ is stated in the equation:

8 $$\:{i}_{Lk\left(t\right)}={i}_{Lk}\left({t}_{2}\right)+{V}_{C1}-\:{V}_{C3}/n\:-{V}_{C2}/{L}_{Lk}\left(t-{t}_{2}\right)$$

Mode 3 ends at $\:{t}_{3}$ when $\:{i}_{D2}=0$.

Mode 4 ($\:{t}_{3}<t<{t}_{4})$: In Fig. 4 (d) starts at $\:{t}_{3}$ when diode $\:{D}_{2}$ is reversed bias ($\:{i}_{D2}=0$) while $\:{D}_{1}$ is still in a conduction state. The$\:{i}_{Lk}$ is linearly decreasing across the body diode in $\:{S}_{2}$.

9 $$\:{i}_{Lk}\left(t\right)={i}_{Lk}\left({t}_{3}\right)+\frac{{V}_{C1}-{V}_{C2}}{{L}_{Lk}}\left(t-{t}_{3}\right)$$

The mode stops at the time $\:{t}_{4}$ when diode $\:{D}_{3}$ if off.

Mode 5 ($\:{t}_{4}<t<{t}_{5}):$ In Fig. 4 (e), the interval begins at $\:{t}_{4}$, when the polarity of the CIN reverses, and energy transfer to the load begins with the $\:{D}_{3}$ off and $\:{D}_{4}$ conducting. $\:{i}_{L1}$ and $\:{i}_{Lm}$ decreases linearly and is expressed as follows:

10 $$\:{i}_{L1}\left(t.\right)={i}_{L1}\left({t}_{4.}\right)+{{V}_{in}-V}_{C1}/{L}_{1}(t.-{t}_{4.})$$

11 $$\:{i}_{Lm}\left(t\right)={i}_{Lm}\left({t}_{4}\right)+{{V}_{C1}-V}_{C2}/{L}_{m}(t-{t}_{4})$$

Furthermore, the current through inductors at time $\:{t}_{4}$ are

12 $$\:{i}_{L1}\left({t}_{4}\right)={i}_{L1}\left({t}_{3}\right)+{{V}_{in}-V}_{C1}/{L}_{1}(1-D)T$$

13 $$\:{i}_{Lm}\left({t}_{4}\right)={i}_{Lm}\left({t}_{3}\right)+{{V}_{C1}-V}_{C2}/{L}_{m}(1-D)T$$

Mode 6: ($\:{t}_{5}<t<{t}_{6}):$ Fig. 4 (f), this mode starts at $\:{t}_{5}$ when $\:{S}_{2}$ off and $\:{D}_{1}\:$and $\:{D}_{4}$ on. $\:{C}_{S1,\:}$will be on because of the polarity reversal of the leakage inductor current $\:{i}_{Lk}$ therefore, the drain to source voltage of $\:{S}_{1}$ is zero, at this moment $\:{S}_{1}$ will be ON with ZVS. This mode finishes at the time $\:{t}_{6}.\:$The $\:{V}_{CS}$ and $\:{i}_{LK}$ are stated as:

14 $$\:{V}_{CS}\left(t\right)=\left({V}_{C2}-{V}_{o}\right)\text{cos}\frac{t-{t}_{5}}{\sqrt{{V}_{CS}{L}_{LK}}}+{i}_{LK}\left({t}_{5}\right)\sqrt{\frac{{L}_{LK}}{{C}_{S}}}\text{sin}\frac{t-{t}_{5}}{\sqrt{{L}_{LK}{C}_{S}}}+{V}_{o}$$

15 $$\:{i}_{LK}\left(t\right)={i}_{LK}\left({t}_{5}\right)\text{cos}t-{t}_{5}/\sqrt{{L}_{LK}{C}_{S}}+{V}_{o}-{V}_{Cc}/\sqrt{\raisebox{1ex}{${L}_{LK}$}\!\left/\:\!\raisebox{-1ex}{${C}_{S}$}\right.}{sin}t-{t}_{5}/\sqrt{{L}_{LK}{C}_{S}}\:$$

## Selection criterion

### Selection criterion for voltage gain

As seen from the waveform and utilizing the concepts of volt-second balancing on the inductor $\:{L}_{1}\:$and $\:{L}_{m}$ utilising Eqs. (4), (12), (5), and (13) respectively, yields.

16 $$\:{V}_{in}={V}_{C1}\left(1-D\right)$$

17 $$\:{V}_{C1}={V}_{C2}\left(1-D\right)$$

However, $\:{C}_{3}$ is charged via $\:{L}_{2}$and $\:{L}_{3}$‘s magnetic connection and the voltage across $\:{C}_{3}$ is

18 $$\:{V}_{C3}=n{V}_{C1}$$

Similarly, using Kirchhoff’s voltage theory at the output and simplifying gives.

19 $$\:{V}_{C2}=\frac{{V}_{o}}{1+n}$$

Substituting Eq. (17) into (19) and simplifying yields

20 $$\:{V}_{C1}={V}_{o}\frac{\left(1-D\right)}{\left(1+n\right)}$$

Similarly, by substituting Eq. (16) into (20), the overall VCR is:

21 $$\:\frac{{V}_{o}}{{V}_{in}}=\frac{1+n}{{\left(1-D\right)}^{2}}$$

Considering the effect of $\:{L}_{LK\:}$, the VGN of the suggested converter is expressed as (22)

22 $$\:\frac{{V}_{o}}{{V}_{in}}=\frac{1+n}{{\left(1-D\right)}^{2}(1+2{n}^{2}{f}_{s}\frac{{L}_{LK}}{{R}_{L}}{D}^{2}+2{n}^{2}{f}_{s}\frac{{L}_{LK}}{{R}_{L}}{(1-D)}^{2})}$$

The suggested converter performance regarding VGN is analysed across varying duty cycles and turns ratios, as shown below. These comparisons provide insights into the proposed converter design’s effectiveness in achieving higher voltage gains with increased flexibility for different applications.

**Fig. 5:**
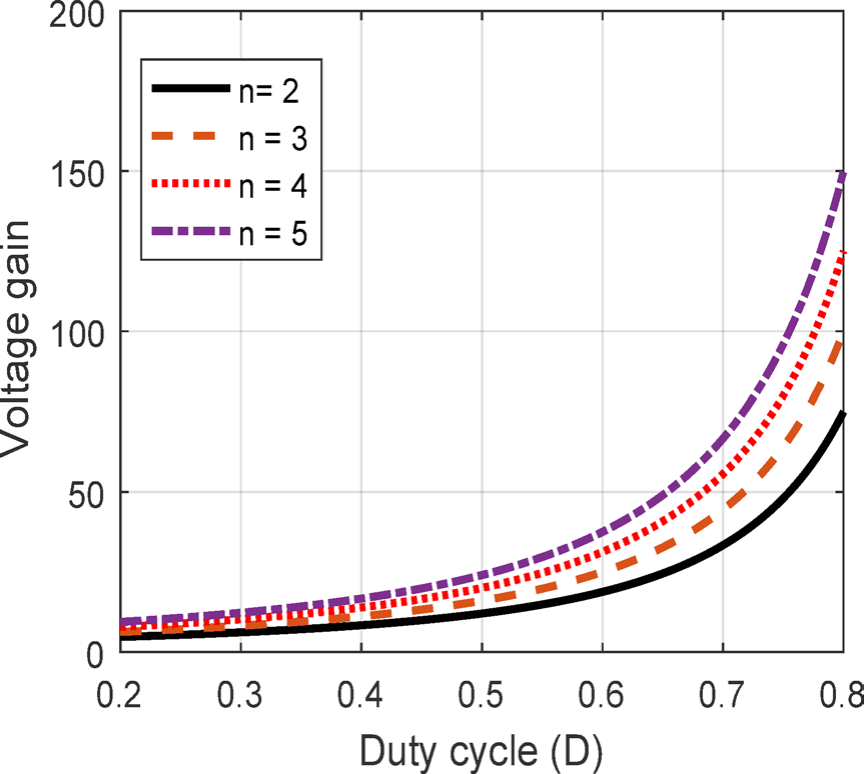
VGN variation with duty cycle.

Figure 5 shows the effect of duty cycle *D* variation on VGN for different n (where $\:n=\:$2,3,4 and 5). When *D* increases, VGN increases exponentially, demonstrating that higher duty cycles can yield significantly greater output voltages. However, this VGN becomes more pronounced at larger values of the turn’s ratio ($\:n$). For instance, with $\:n=5$, the voltage gain reaches almost 150 at a duty cycle of 0.8, far surpassing the VGN achieved with lower turn ratios. This trend highlights the proposed QBC capability to adapt to high-gain requirements, making it particularly advantageous for applications where high output voltages are essential, such as in renewable energy systems.

**Fig. 6:**
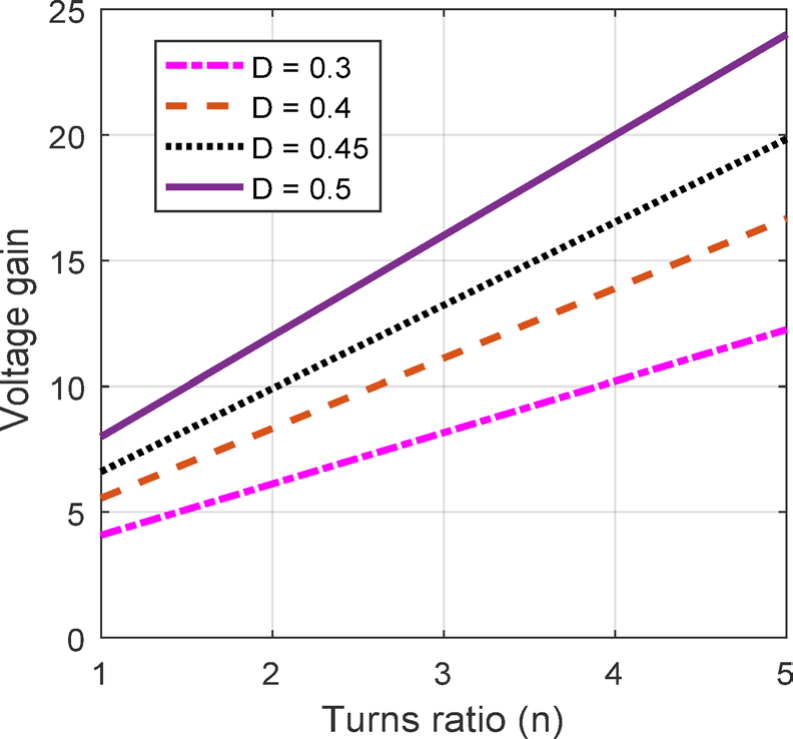
VGN variation with turns ratio.

Figure 6 illustrates the connection between VGN and the turns ratio *n* at duty cycles 0.3, 0.4, 0.45, and 0.5 respectively. This shows a linear voltage gain increase as the turn ratio increases. Higher duty cycles, such as $\:D$ = 0.5, indicate a greater sensitivity to turn ratio changes. This linear gain response based on the *n* provides a proper design parameter that enables them to achieve desired voltage levels by adjusting *n* without necessarily pushing the duty cycle to higher levels, which could otherwise introduce EMI related issues due to diodes (Rr) loss. The combination of these two graphs demonstrates the flexibility and adaptability of QBC design. By modifying the *D* and the ratio, the converter can accomplish high VGN in a controlled and efficient manner.

**Fig. 7:**
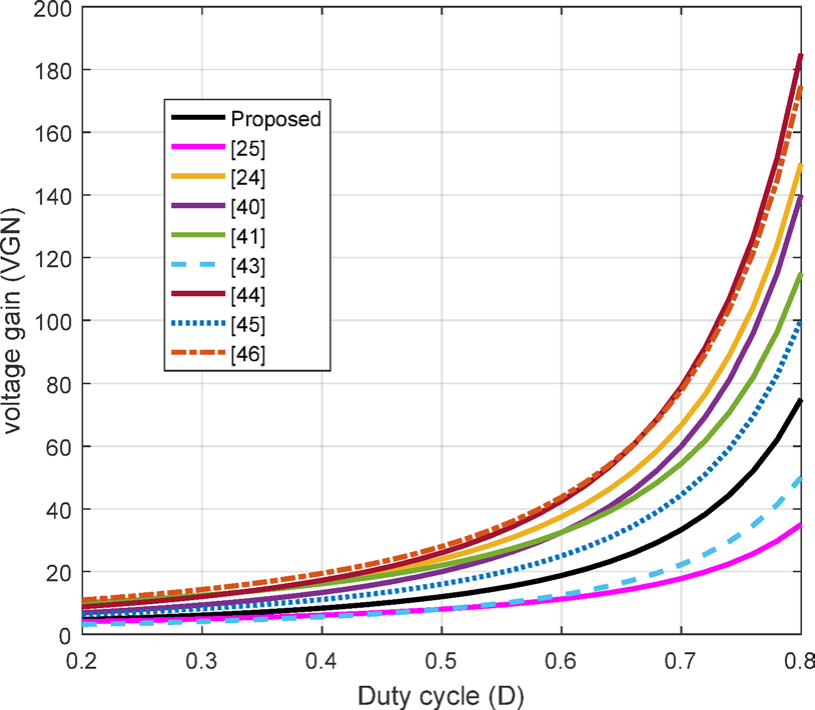
Comparison of VGN with multi-converters.

Figure 7 compares the VGN of the proposed QBC with several converters reported in previous research, including those from^24,25,40,41,43–45^, and^46^, across varying duty cycles. Although some of these converters have high VGN when compared to the proposed converter, the VSS on semiconductors devices for converters in converters^25,40,43^, and^46^ is higher. Furthermore, the semiconductor devices in^25,40,43–45^ are operated under hard switching conditions while^24,46^ are partly operated under soft-switched. In addition, converters reported in^24,25,45,46^ show high number of component counts. Hence the degradation in efficiency.

**Fig. 8:**
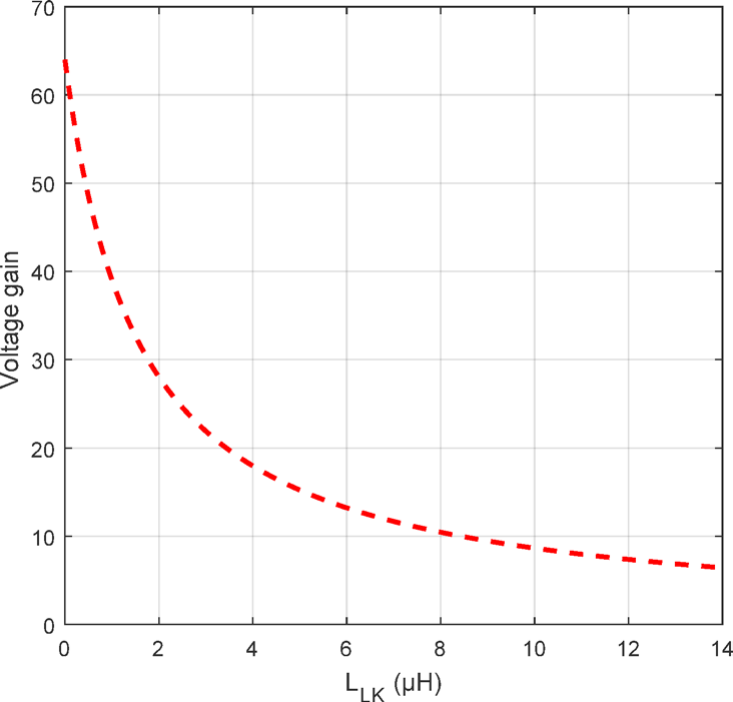
Effect of $\:{L}_{LK\:}$ on the converter gain.

The proposed QBC, represented by the solid black line, demonstrates a significantly higher voltage gain than^26,43^. This enhanced performance is attributed to the integration of coupled inductors and an active clamp circuit, which effectively minimizes energy losses and improves efficiency. In contrast with the aforementioned converters the proposed converter exhibits moderate voltage gains, fully soft switching operation, low component count and low VSS on devices. These results highlight the superior efficiency and high-gain capability of the proposed QBC compared to existing designs. In addition, the effects of $\:{L}_{LK\:}$on the converter gain is illustrated in Fig. 8. An increase in the$\:{L}_{LK\:}$ reduces the VCR showcasing and additional energy loss which also affects on overall efficiency.

### Selection criterion for components

This part illustrates the design of the proposed QBC. To operate it in CCM, selecting an appropriate switching frequency and component values is essential to ensure the proposed converter’s adequate performance.

1. Selection of inductor and capacitor.

Value of $\:{L}_{1}$ chosen to guarantee that the converter will always run in a CCM. Considering the nominal output power and the inductors’ ripple current and assuming the extreme current ripple rate of $\:L$ is $\:{x}_{L}$ then the following equations should be satisfied^36^.

23 $$\:\varDelta\:{i}_{L}\le\:{x}_{L}{i}_{L\_ave}$$

Then inductor $\:{L}_{1}$ is expressed as:

24 $$\:{L}_{1}\ge\:\frac{{V}_{in}DT}{\varDelta\:{i}_{L1}}$$

Considering the position of the CIN i.e. placed after the input; this does not require a minimal input current ripple in its construction. Hence, magnetising inductor values are obtained using the expression:

25 $$\:{L}_{m}\ge\:\frac{D{V}_{C1}}{\varDelta\:{I}_{Lm}{f}_{s}}$$

Similarly, the value of $\:{C}_{o}$ can be ascertained using the capacitor voltage ripple Eqs. (26) and (27) respectively^37^.

26 $$\:\varDelta\:Q=C\varDelta\:{V}_{C}$$

27 $$\:{C}_{o}=\frac{D{V}_{o}}{\varDelta\:{V}_{co}R{f}_{s}}$$

Moreover, $\:{C}_{1},\:{C}_{2},\:{C}_{3}$ are determined as shown in:

28 $$\:\left\{\begin{array}{c}{\:C}_{1}=\frac{{I}_{L1}D}{\varDelta\:{V}_{C1}{f}_{s}}\\\:{\:\:\:\:C}_{2}=\frac{{I}_{o\left(\text{m}\text{a}\text{x}\right)}D}{\varDelta\:{V}_{C2}{f}_{s}}\\\:\:\:\:\:{C}_{3}=\frac{{I}_{o\left(\text{m}\text{a}\text{x}\right)}D}{\varDelta\:{V}_{C3}{f}_{s}}\end{array}\right.$$

2) Condition for soft switching operation.

As noticed Fig. 4(f), since the $\:{i}_{LK}$ is negative, $\:{C}_{s}$ is discharged by $\:{i}_{LK}$. The $\:{V}_{CS}\:$should be zero before the end of this mode to achieve ZVS. For this condition to be achieved, the energy in the $\:{L}_{LK\:}$ must be greater than the energy stored in the $\:{C}_{S}$. Therefore, for ZVS turn-on, the inequality condition of Eq. (29) must be satisfied^38,39^.

29 $$\:\frac{1}{2}{L}_{Lk}{i}_{LLk}^{2}\ge\:{\frac{1}{2}C}_{S}{v}_{Sc}^{2}$$

30 $$\:{L}_{Lk}\ge\:\frac{{C}_{S}{v}_{Sc}^{2}}{{\left({i}_{LK}\right)}^{2}}$$

## Determination of stress across components

The stress on a converter over a switching period can be classified as current and VSS across the devices. This is used to analyse the reliability and efficiency of a converter. When $\:{S}_{1}$ and $\:{S}_{2}$ are turned off, the voltage in $\:{S}_{1,\:}\:{S}_{2}$ is clamped at $\:{v}_{C1}$, hence, the VSS of the$\:{S}_{1}$ and $\:{S}_{2}$ is obtained as:

31 $$\:{V}_{s1}={V}_{s2}={V}_{C1}={V}_{o}\left(1-D\right)/\left(1+n\right)$$

Similarly, VSS of diodes are approximated as follows:

32 $$\:\left\{\begin{array}{c}{v}_{D1}={v}_{D2}\cong\:\frac{{V}_{in}}{1-D}\:\\\:{v}_{D3}\cong\:{v}_{o}-{v}_{C1}\:\:\:\:\\\:{v}_{D4}\cong\:{v}_{o}\:\:\:\:\:\:\:\:\:\:\:\:\:\:\:\:\end{array}\right.$$

The maximum current stress across the $\:{S}_{1}$ and $\:{S}_{2}$ and diodes are roughly equal to:

33 $$\:\left\{\begin{array}{c}{i}_{dS1\_max}={i}_{dS2\_max}\cong\:{i}_{L1\_max}+{i}_{Lm\_max}\\\:{i}_{D1}={i}_{D2}\cong\:{i}_{o\_max}/\left(1-D\right)\:\:\:\:\:\:\:\:\:\:\:\:\:\:\:\:\:\:\:\:\:\:\:\:\:\:\:\:\:\:\:\:\:\:\:\:\:\:\:\:\:\:\:\\\:{i}_{D2}={Di}_{o\_max}/{\left(1-D\right)}^{2}\:\:\:\:\:\:\:\:\:\:\:\:\:\:\:\:\:\:\:\:\:\:\:\:\:\:\:\:\:\:\:\:\:\:\:\:\:\:\:\:\:\:\:\:\:\:\:\:\:\:\:\:\\\:{i}_{D3}={i}_{Lm\_max}\:\:\:\:\:\:\:\:\:\:\:\:\:\:\:\:\:\:\:\:\:\:\:\:\:\:\:\:\:\:\:\:\:\:\:\:\:\:\:\:\:\:\:\:\:\:\:\:\:\:\\\:{i}_{D4}={i}_{o\_max}\:\:\:\:\:\:\:\:\:\:\:\:\:\:\:\:\:\:\:\:\:\:\:\:\:\:\:\:\:\:\:\:\:\:\:\:\:\:\:\:\:\:\:\:\:\:\:\:\:\:\:\:\:\end{array}\:\right.$$

**Fig. 9:**
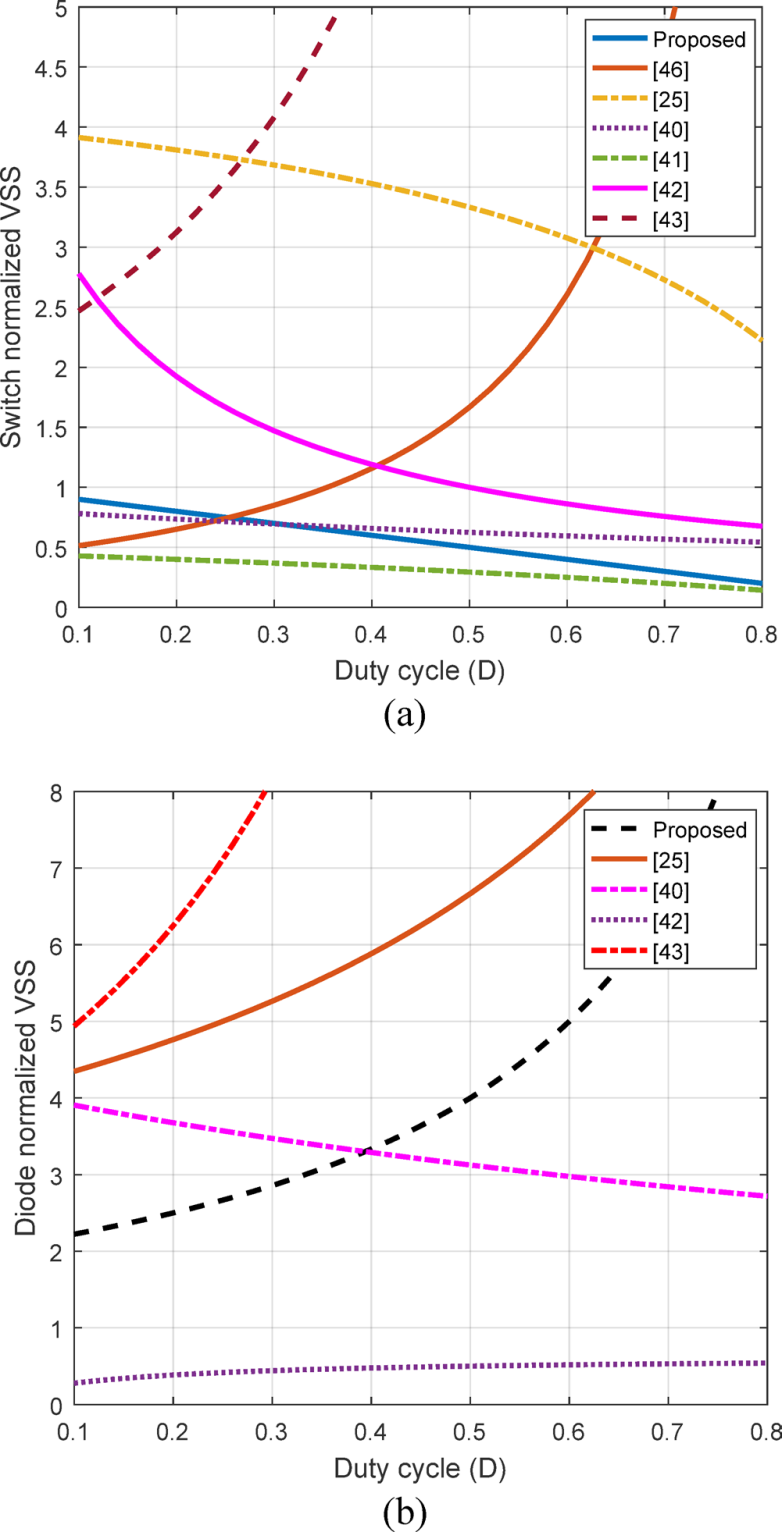
Comparison of normalized VSS variation with D on semiconductor devices for the proposed converter and converters reported in^25,40–43,46^ (a) switch VSS (b) diodes VSS.

To help present a generalized behaviour across operating conditions by reducing dependence on absolute scales, a normalized VSS across semiconductor devices is presented. For the purpose of benchmarking, the suggested converter and converters reported in^25,40–43,46^ are simulated under the same turn ratio and the variation of VSS with $\:D$ is depicted in Fig. 9. Figure 9 (a) shows the normalize VSS variation with $\:D$ for the power switch. The proposed method, indicated by the solid blue line, shows a decreasing trend in voltage stress as the duty cycle increases, ultimately exhibiting lower VSS than the other methods across the range. On the other hand, the normalized VSS for the diode is depicted in Fig. 9 (b). The proposed topology VSS (dotted black line) demonstrates lower voltage stress in comparison with converters in^25,40,43^ particularly at lower duty ratio which is below 0.4. This suggests that the proposed method provide better performance by reducing voltage stress, which improves efficiency and longevity in applications that involve varying duty cycles.

## Experimental and simulation results demonstration

The simulation results are shown here, along with the outcomes of practical experiments, which will be discussed in greater detail.

### Simulation results

**Fig. 10:**
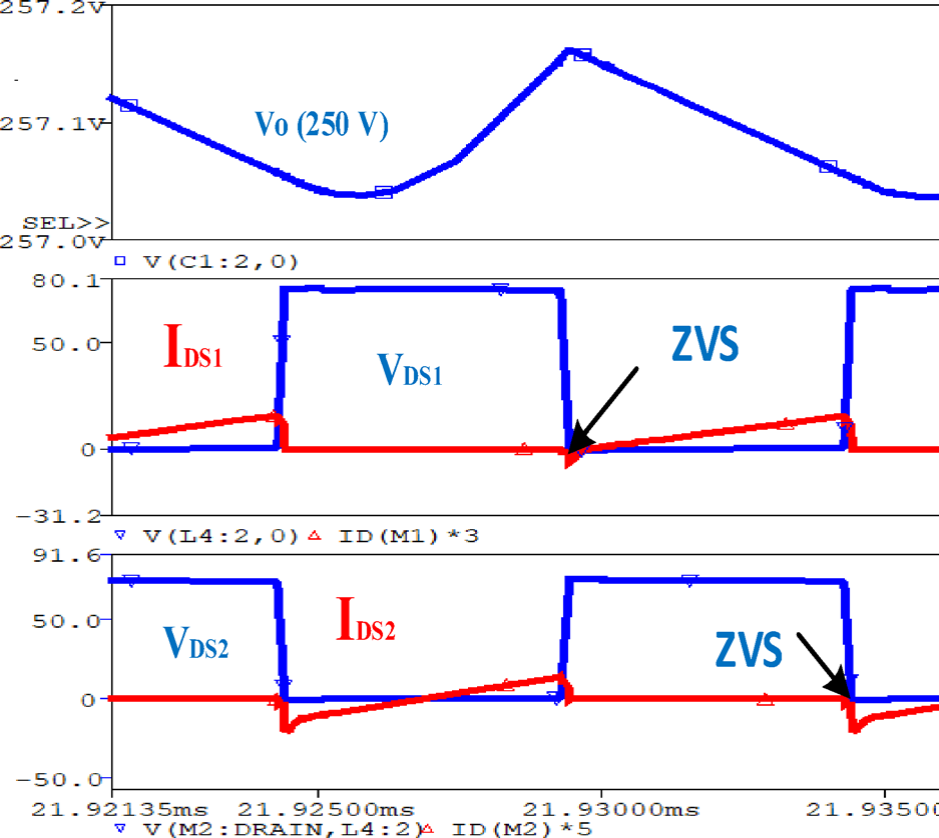
Simulation for switch $\:{S}_{1}$and $\:{S}_{2}\:$with the output $\:{V}_{\text{O}}.$.

The PSpice simulation result shown in Fig. 10 illustrates the behaviour of a switching circuit along with the corresponding $\:{V}_{O\:}.$ The drain-source voltage across $\:{S}_{1}$ and $\:{S}_{2}$ ($\:{V}_{DS1}$ and $\:{V}_{DS2})$, and the corresponding drain-source currents $\:{I}_{DS1}\:$and $\:{I}_{DS2}$ are illustrated. The $\:{V}_{O\:}$of 250 V is shown. The results demonstrate the ZVS ability of the switches at turn on instants.

**Fig. 11:**
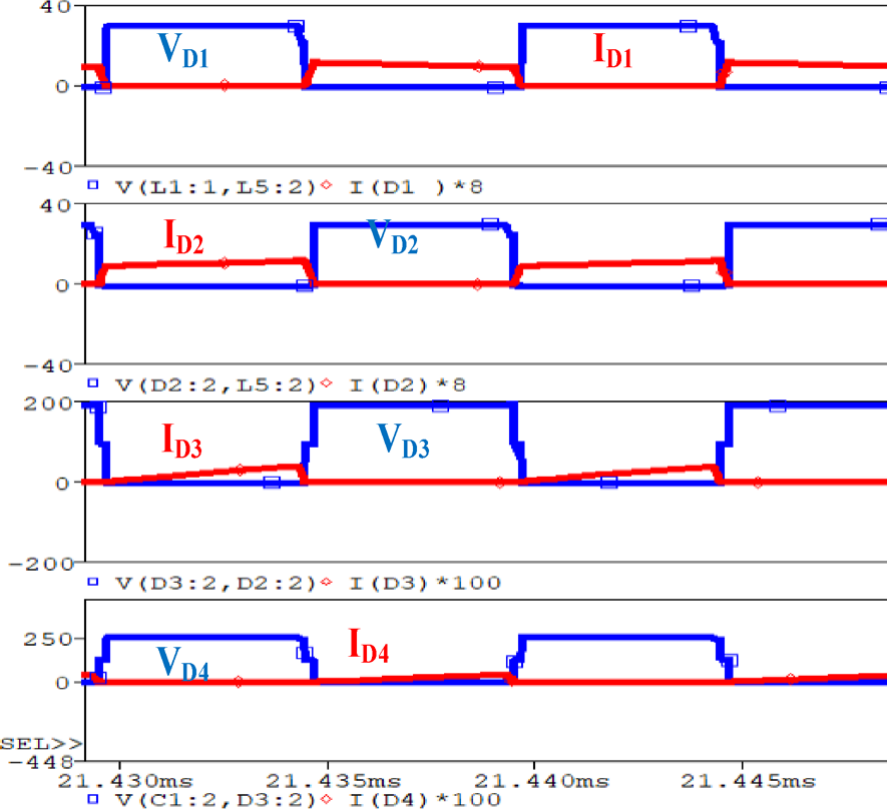
Simulation for $\:{D}_{1\:},\:{D}_{2},\:{D}_{3}\:$and $\:{D}_{4}.$.

In Fig. 11, the simulation illustrates the operation of four diodes in a circuit designed in order to attain ZVS and ZCS. Each plot shows the $\:{V}_{D\:}$ and $\:{I}_{D\:}$for all diodes respectively. The blue waveforms represent the voltages, while the red waveforms represent the currents. In ZVS, the diodes are ON when the voltage across them is near zero, reducing switching losses. Similarly, in ZCS, the diodes are OFF when the flowing through them approaches zero, minimizing switching stresses.

### Results of the experiment

**Fig. 12:**
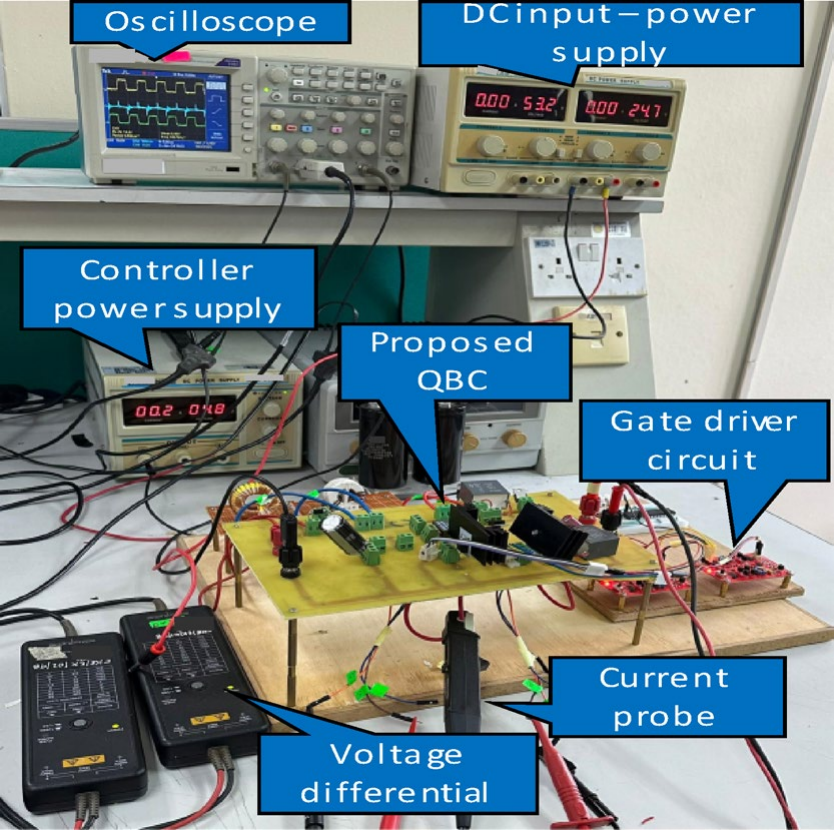
Experimental set-up of the QBC.

**Fig. 13:**
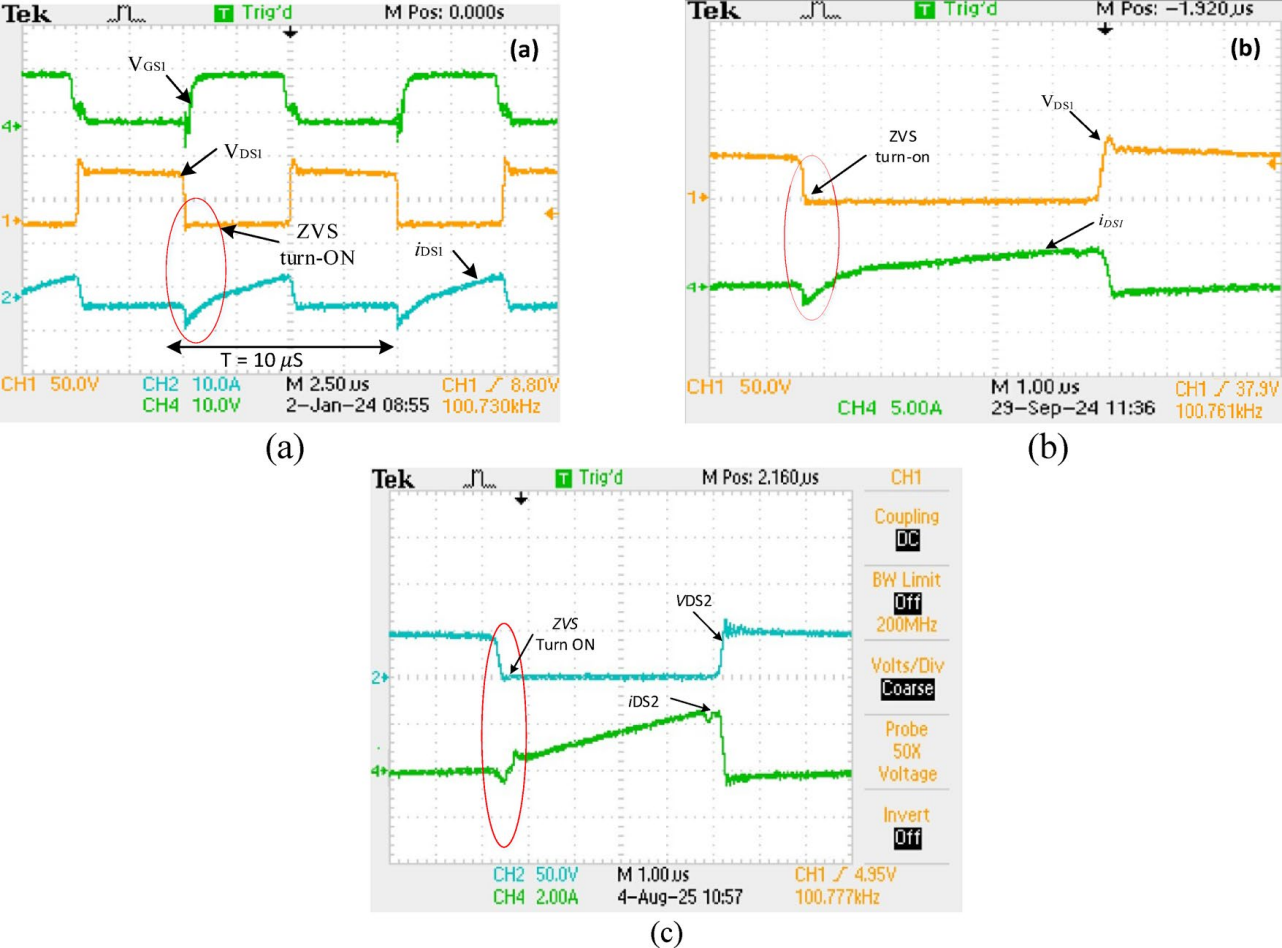
Experimental waveforms of voltage through $\:{S}_{1}$ ($\:{V}_{DS1}$) and ($\:{i}_{DS1}$) across switch $\:{S}_{1}$ at *D* = 50% (a) at 100% load (b) at 50% load (c) at 20% load.

Table 1 highlights the critical parameters considered for the experimental and simulation validation of the prototype suggested converter, whereas Fig. 12 depicts the prototype converter’s experimental configuration. Figures 13 (a, b, and c) show the result of$\:{V}_{DS1,}$ and $\:{I}_{DS1,}$ across and through switch $\:{S}_{1}$at 50% duty cycle. There are three operating conditions: (a) at 100% load and (b) at 50% load and (c) 20% load. In both cases, the $\:{V}_{DS,}$ and $\:{I}_{DS,}$ traces demonstrate ZVS turn-on, where the voltage through the$\:\:{S}_{1}$ decreases to zero before it is ON, reducing switching losses and enhancing efficiency. ZVS turn-on occurs within a 10 µs period at full load. Similarly, this operation condition confirms the suggested converter soft-switching functionality at varying load conditions.

**Table 1:** Selected components for experimental hardware.

Parameters	Specifications
$\:{V}_{in}$	$\:24\:V$
$\:{V}_{o}$	$\:250\:V$
$\:f$	$\:100\:kHz$
Inductors	$\:{L}_{1}=155\:\mu\:H\:\:$ $\:{L}_{m}=380\:\mu\:H\:$ $\:{L}_{Lk}=10\:\mu\:H\:$
Capacitors	$\:{C}_{1}=200\:\mu\:F\:$ $\:{C}_{2}=2\:\mu\:F$ $\:{C}_{3\:}=5\:\mu\:F$ $\:{C}_{o\:}=5\:\mu\:F\:\:$
Diode	STTH15L06D; $\:{V}_{F}=0.95\:V$
MOSFET	IRF150P220AKMA 1 (150 V, 2.3 mΩ)

**Fig. 14:**
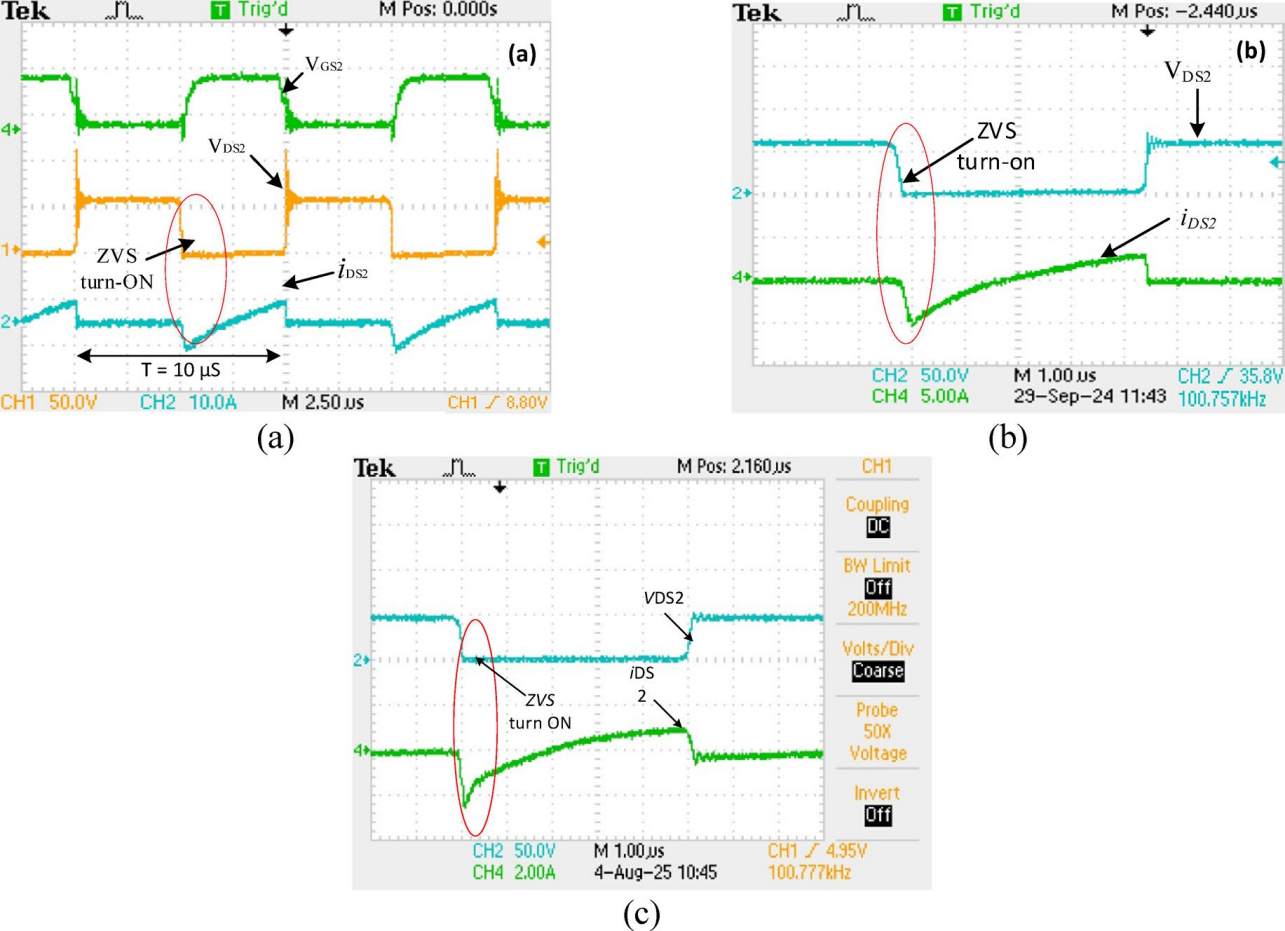
Experimental waveforms of voltage through $\:{S}_{2}$ ($\:{V}_{DS2}$) and ($\:{i}_{DS2}$) across switch $\:{S}_{2}$ at *D* = 50% (a) at100% load (b) at 50% load (c) at 20% load.

**Fig. 15:**
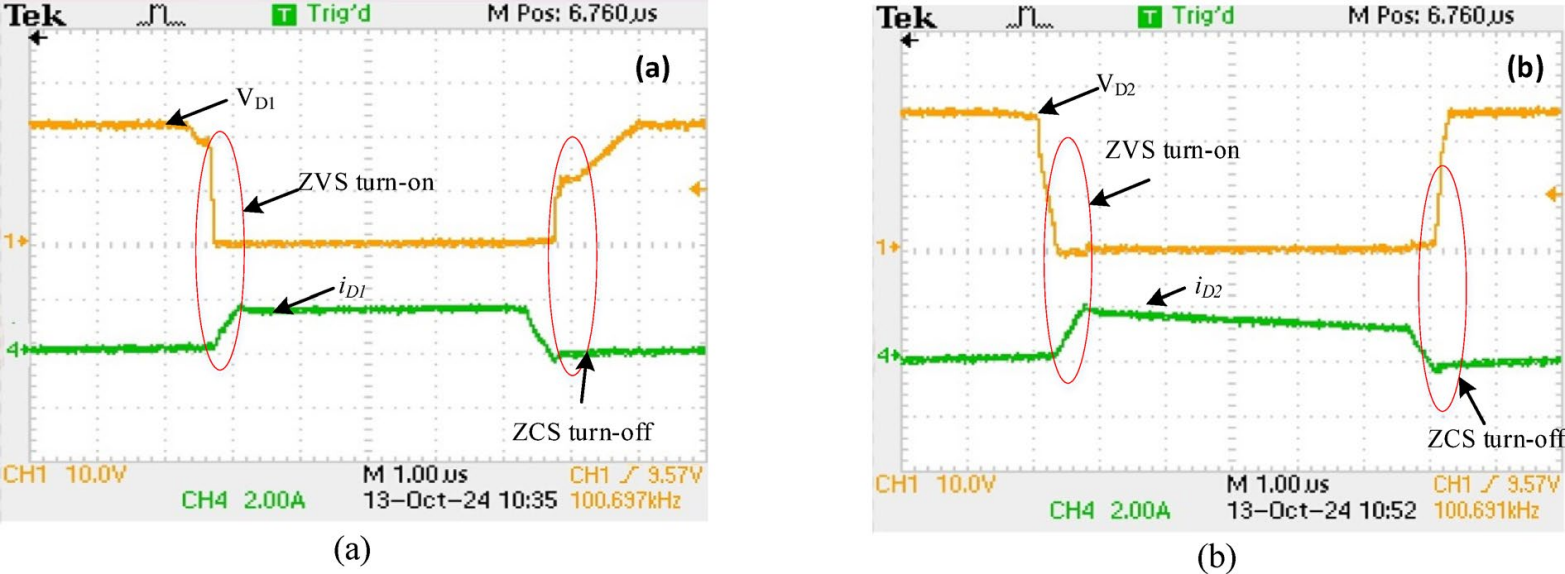
Results of experimental of the diodes’ voltage with current (a) diode $\:{D}_{1}$ (b) diode $\:{D}_{2}$.

Figure 14 displays the waveforms of $\:{V}_{DS2}$ and $\:{I}_{DS2}$ across$\:{S}_{2,}$ ZVS operation is demonstrated, at 100% load, 50%load and at 20% load as seen in Figs. 14 (a, b and c) respectively. In both cases, the ZVS turn-on is highlighted, where the voltage through the $\:{S}_{2,}$ goes down to zero before it turns on, decreasing switching losses. From the waveform, it can be noticed that ZVS turn-on is realized over a period of 10 µs at all load condition, which demonstrates that ZVS is consistently achieved across different load conditions. The ZVS operation under varying loads is crucial for enhancing the efficiency and longevity of the switching process. Similarly, due to an active network, the VSS on the switches is reduced and agrees with the analytical value expressed in (27). Figures 15 (a, b) show the $\:{V}_{D1}\:$, $\:{V}_{D2}$ and $\:{i}_{D1\:}$, $\:{i}_{D2}\:$across diodes$\:\:{D}_{1}$,$\:{D}_{2}$ respectively. In both cases, the ZVS turn-on is shown, where the diode voltage decreases to zero before current flows, reducing switching losses at turn-on.

Two diodes function at ZVS turn-on and ZCS turn-off, optimizing efficiency by reducing switching losses in both stages.

**Fig. 16:**
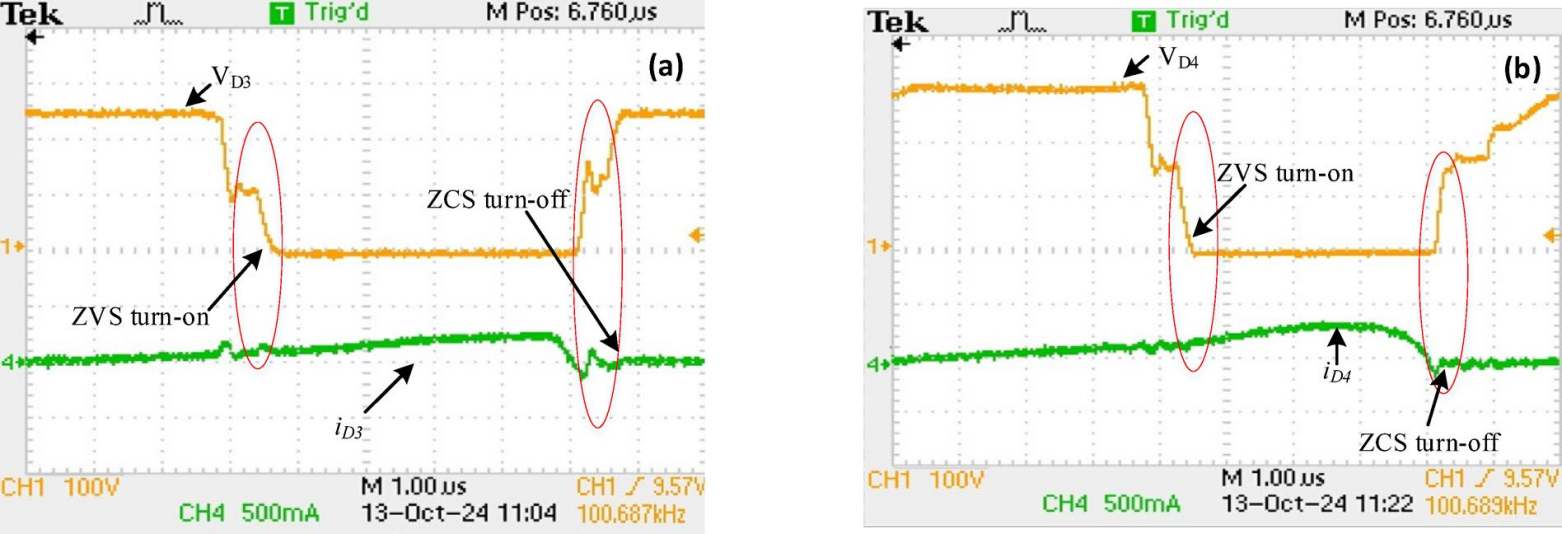
Experimental results in the diodes’ voltage with current (a) diode $\:{D}_{3}$ (b) diode $\:{D}_{4}$.

**Fig. 17:**
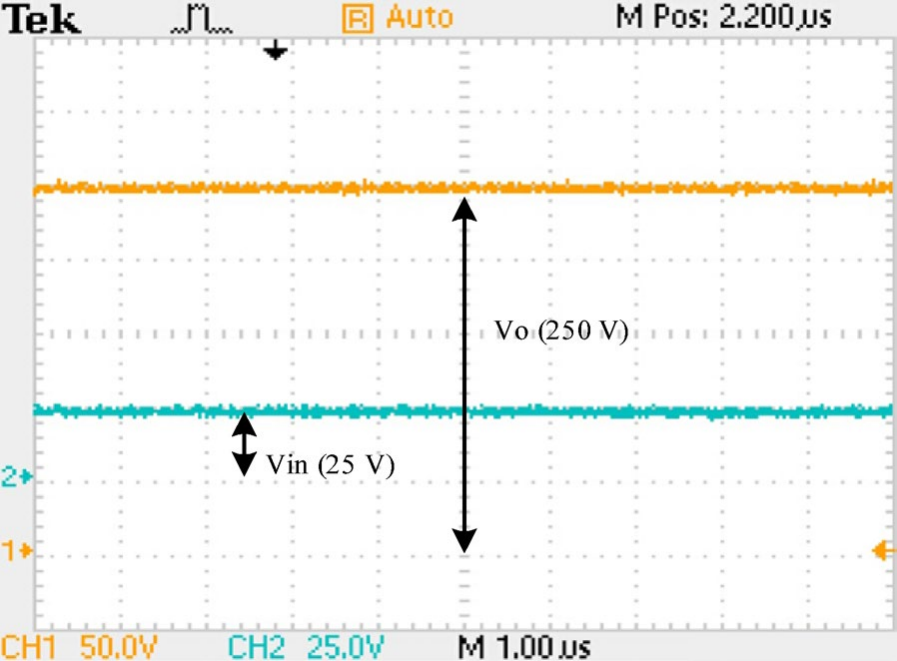
Full load experimental voltage waveform of the QBC.

Similarly, Figs. 16 (a, b) show diodes $\:{D}_{3}$ and $\:{D}_{4}\:$operating under ZVS/ZCS. All the four diodes operate under ZVS/ZCS, eliminating the reverse recovery losses. Figure 17 shows that at full-load the converter’s ($\:{V}_{O\:}$) is 250 V at ($\:{V}_{in}$) = 24 V.

## Practical application and suitability

The proposed quadratic boost converter QBC demonstrates a high degree of suitability for several.

modern power-electronic applications, particularly in renewable systems, EV charging stations, and DC microgrids, where both efficiency and reliability are paramount. In PV arrays, fuel cells, and small wind turbines, maintaining a smooth and CCM is essential to extend the lifetime and operational stability of the source. The use of an input inductor in the proposed topology ensures a CCM, effectively mitigating the harmful effects of current pulsations on PV modules. This feature directly enhances the system’s mean time before failure (MTBF) and contributes to improved power quality when integrating intermittent renewable energy into the grid. Moreover, the converter’s ability to achieve high VGN at relatively low $\:D$ reduces the control stress on the switches, which is particularly beneficial in high-power installations.

In addition to its CCM characteristic, the proposed converter provides low VSS on the active switches and achieves soft-switching operation via ZVS, thereby reducing switching losses and EMI. Compared with similar topologies, such as the design in^24^, the proposed QBC reaches comparable high-gain performance without requiring excessively large duty cycles. While the topology in^24^ also achieves ZVS, it does so by employing an auxiliary resonant network, which inevitably increases both circuit complexity and manufacturing cost. Similarly, the design in^25^ attempts to improve voltage stress performance by further increasing the duty cycle; however, this approach aggravates the (Rr) behavior of the diodes, leading to additional power loss and potential reliability issues.

Converters intended for EV charging stations, fuel cell systems, and DC microgrids have also been explored in^42,44^. Although these designs achieve high voltage gains, their reliance on three-winding coupled inductors (CI) and multi-stage boosting configurations increases the converter footprint, weight, and cost. In contrast, the proposed topology maintains a compact structure while delivering competitive performance metrics, making it advantageous for space- and cost-sensitive installations.

## Comparative analysis

### Loss analysis and efficiency

**Fig. 18:**
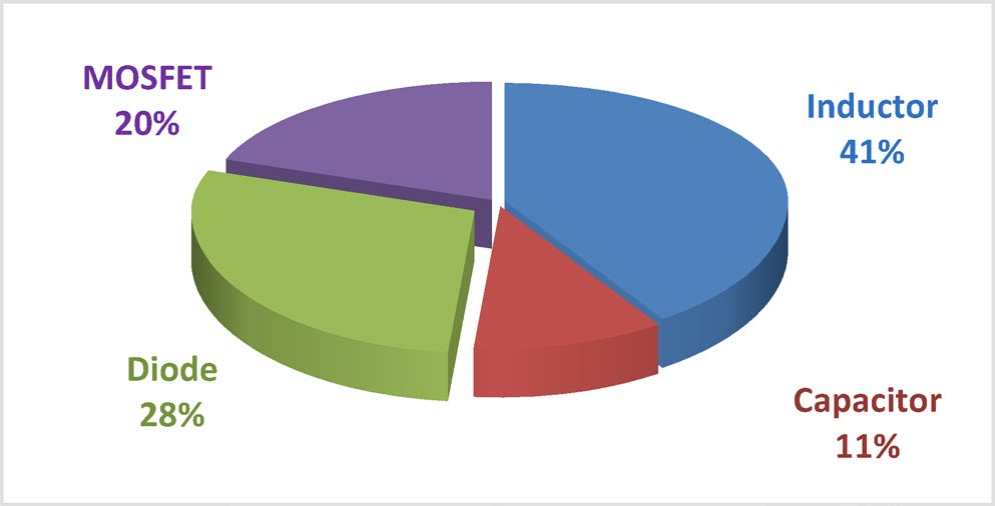
Components loss distribution for the QBC.

**Fig. 19:**
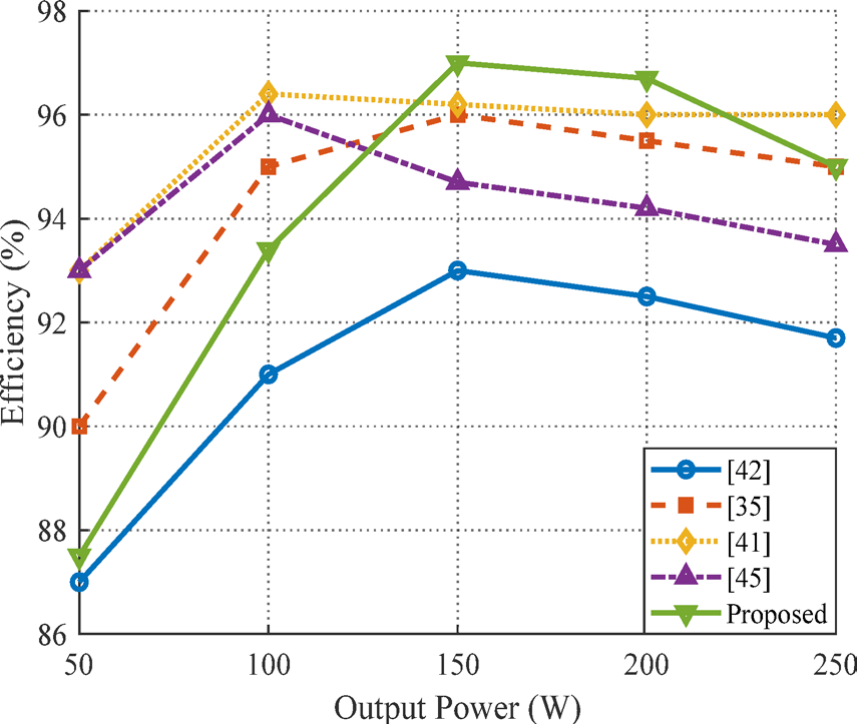
Efficiency of the proposed QBC.

Loss analysis and efficiency of the suggested converter are evaluated for a fair comparison with comparable converters reported in the literature. Loss analysis on individual components is necessary to verify the converter performance in real-time application. Capacitor ripple voltage and inductor ripple currents are neglected for ease of analysis. The loss across $\:{S}_{1,\:}\:{S}_{2}\:$consists of switching loss at turn-off ($\:{P}_{sw\_off})\:$and conduction ($\:{P}_{sw-cond})\:$loss given by:

34 $$\:\left\{\begin{array}{c}{P}_{sw\_off}=\frac{1}{2}{V}_{ds}{I}_{ds}{t}_{f}{f}_{s}\:\\\:{P}_{sw-cond}={i}_{rms}^{2}{R}_{d{s}_{on}}\end{array}\right.$$

where $\:{R}_{ds\_on}$ is the drain-source resistance and $\:{i}_{rms}$ is the root mean square current of the switch, $\:{V}_{ds}$ and $\:{I}_{ds}$ are the drain-source current of the switch, while $\:{t}_{f}$ and $\:{f}_{s}$ are the switch falling time and$\:{f}_{s\:}$ respectively. The diode is ON and off under ZVS/ZCS; hence, conduction is the dominant loss across the diode. Diode conduction loss ($\:{P}_{D-cond})$, winding loss of the inductor ($\:{P}_{L-win})$, and capacitor losses ($\:{P}_{C1})$ are computed using:

35 $$\:\left\{\begin{array}{c}{P}_{D-cond}=\frac{1}{2}{V}_{F}{I}_{av}D\:\\\:{P}_{L-win}={i}_{rms}^{2}{R}_{DC}\\\:{P}_{C1}={i}_{rms}^{2}ESR\:\:\:\:\:\end{array}\right.$$

where $\:{V}_{F},\:{I}_{av}\:\:$are the diode forward voltage drop and average current, and $\:{R}_{DC}$ and$\:\:ESR$ are the DC resistance of the inductor winding and the capacitor’s equivalent series resistance, respectively. The efficiency is determined by considering the losses across individual components as

36 $$\:Efficiency\:=\frac{{P}_{o}}{{P}_{o}+{P}_{T\left(loss\right)}}$$

where $\:{P}_{o}$ is the output power and $\:{P}_{T\left(loss\right)}$ is the sum of the losses expressed in (30) and (31). The loss distribution of individual components obtained using expressions (30) and (31) are displayed in Fig. 18. It is evident that, at 41%, the inductor causes the largest loss, followed by the diode at 28%, the MOSFET at 20%, and the capacitor at 11%. This breakdown highlights the inductor and diode as primary sources of loss within the converter, which is crucial for targeted optimization in future designs. Figure 19 shows the efficiency of the suggested converter in comparison to existing designs referenced in^35,41,42^, and^45^ across a variety of output power levels. The suggested converter exhibits a peak efficiency close to 97% at around 150 W and maintains higher efficiency than most references, particularly at higher output powers. This performance demonstrates the converter’s effectiveness in minimizing losses and delivering superior efficiency in high-power applications.

Table 2 compares the converter design with multiple additional converters documented in the literature, focusing on various performance and design parameters. The key metrics include the normalized (dimensionless or scaled form) VGN, switching stress, diode stress, power rating, efficiency, soft-switching capability, switching frequency, and output voltage range. Each row represents a different converter configuration, highlighting efficiency and stress management trade-offs. Notably, the proposed converter demonstrates enhanced efficiency (97%) and advanced soft-switching features (ZVS/ZCS) compared to others, alongside competitive voltage gain and stress characteristics, showcasing its improved performance for practical applications.

**Table 2:** A comparison of the suggested QBC with some selected converters reported in the literature.

Ref		S/D/L/CI/C/T	Normalized VGN	Switch VSS	Diode VSS	Power (W)	Effi. (%)	SS	$\:{\varvec{f}}_{\varvec{s}}$ (kHz)	$\:{\varvec{V}}_{\varvec{i}},{\varvec{V}}_{\varvec{o}}$ (V)
^42^		2/6/1/1^2W^/6/16	$\:1+2Nd/{d}^{2}$	$\:{V}_{o}/1+2nd$	$\:{\text{V}}_{\text{o}}\text{d}/1+2\text{n}\text{d}$	250	93	ZVS/ ZCS	100	(30–50), 360
^44^		1/5/1/1^3W^/4/12	$\:\frac{\left(1+2n\right)+d(1+n)}{{\left(1-d\right)}^{2}}$	$\:\frac{1}{\left(1+2n\right)+d(1+n)}$	$\:\frac{2\text{n}+\text{d}}{\left(1+2\text{n}\right)+\text{d}(1+\text{n})}$	400	94.5	NO	40	25, 400
^24^		2/4/1/1^2W^/4/14	$\:\frac{2+n+m}{{\left(1-d\right)}^{2}}$	$\:\frac{{V}_{o}}{G{\left(1-d\right)}^{2}}$	$\:\frac{3+\text{n}+\text{m}}{2+\text{n}+\text{m}}$	150	--	ZVS/ ZVS	100	48, 650
^45^		4/10/6/-/5/25	$\:4/{\left(1-d\right)}^{2}$	$\:\frac{{V}_{o}}{4}$	$\:\frac{{\text{V}}_{\text{o}}}{2}$	660	95.8	Hard	60	(20–36) 600
^40^		1/5/1/1/4/12	$\:\frac{2+n+nd}{{\left(1-d\right)}^{2}}$	$\:\frac{{V}_{o}}{2+n+nd}$	$\:\frac{\left(1+\text{n}\right){\text{V}}_{\text{o}}}{2+\text{n}+\text{n}\text{d}}$	160	92.4	Hard	--	20, 400
^25^		2/5/1/1^3W^/4/13	$\:\frac{n\left(1-d\right)+1}{{\left(1-d\right)}^{2}}$	$\:\frac{n\left(1-d\right)}{n\left(1-d\right)+1}{V}_{o}$	$\:\frac{\text{n}}{\text{n}\left(1-\text{d}\right)+1}{\text{V}}_{\text{o}}$	200	96	Hard	100	48, 380
^26^		1/5/1/1^3W^/4/12	$\:\frac{2+n2+n3}{\left[1-d\left(1+n3\right)\right]\left(1-d\right)}$	$\:\frac{{V}_{o}}{2+n2+n3}$	$\:\frac{1+\text{n}2+\text{n}3}{2+\text{n}2+\text{n}3}{\text{V}}_{\text{o}}$	200	96	ZCS	50	25, 400
^46^		1/7/1/1^2W^/6/16	$\:3+2n/{\left(1-d\right)}^{2}$	$\:{V}_{o}/3+2n$	$\:\text{n}{\text{V}}_{\text{o}}/3+2\text{n}$	250	94.1	ZCS	50	(16, 20), 400
^41^		2/4/1/1^2W^/4/12	$\:\frac{3+2n-d(1+n)}{{\left(1-\text{d}\right)}^{2}}$	$\:\frac{(1-D){V}_{o}}{3+2n-\text{D}(1+n)}$	$\:\frac{\left[2+2\text{n}-\text{d}(1+\text{n})\right]{\text{V}}_{\text{o}}}{3+2\text{n}-\text{d}(1+\text{n})}$	200	96.1	ZCS	50	25, 400
^43^		1/5/3/-/3/12	$\:2/{\left(1-d\right)}^{2}$	$\:2{V}_{in}/{\left(1-d\right)}^{2}$	$\:2{\text{V}}_{\text{i}\text{n}}/{\left(1-\text{d}\right)}^{2}$	130	--	Hard	50	20, 195
Prop.		2/4/1/1^2W^/4/12	$\:1+n/{\left(1-d\right)}^{2}$	$\:(1-d){V}_{o}/(1+n)$	$\:{\text{V}}_{\text{i}\text{n}}/1-\text{d}$	250	97	ZVS/ ZVSZCS	100	24, 250

### Sensitivity analysis of efficiency

To evaluate the robustness of the suggested converter, a sensitivity analysis of efficiency with respect to the main design and operating parameters was performed. The analysis considered the effect of $\:D$, $\:n\:$and load variation (R). The results show that efficiency is highly dependent on the $\:D$. Within the practical operating range of 0.3 ≤ D ≤ 0.6, the proposed converter consistently achieves efficiency above 95%. Beyond this range, particularly when D approaches 0.7 or higher, conduction losses in the MOSFETs and reverse recovery effects in the diodes become more significant, leading to a gradual reduction in efficiency, which is consistent with the switching and conduction losses already highlighted in Fig. 18.

The influence of the $\:n$ of the CINwas also examined. Increasing n improves the voltage gain but introduces additional winding resistance, which slightly increases copper losses. Nevertheless, the converter maintains efficiency above 94% for the investigated values of n, confirming the design’s ability to achieve high gain without severely compromising performance. Load variation was further studied to reflect real-world operating conditions. At nominal load (250 W), the peak efficiency reaches approximately 97%, as shown in Fig. 19, while even at light load (20% of rated power) efficiency remains above 93%. This demonstrates the capability of the converter to handle dynamic load changes effectively.

Overall, this sensitivity study confirms that the suggested QBC maintains high efficiency across a wide range of $\:D$, load conditions, and turns ratios. This characteristic enhances its suitability for renewable energy and electric vehicle applications, where operating points are often variable.

## Conclusion

The suggested high VGN, high-efficiency QBC presented in this study, a new method for applying renewable energy is presented. By employing a coupled inductor and an active clamp network, this converter provides VGN without requiring extreme $\:D$, addressing the Limitations of traditional converters. Notably, the design facilitates ZVS for the energy switches and ZCS for the diodes, minimizing switching losses and remarkably enhancing efficiency. This soft-switching operation, combined with low VSS through semiconductor devices, ensures improved reliability and longevity for the system. Furthermore, a continuous input and output current waveform and the common ground connection between input and output simplify integration with other system components in renewable energy systems. Through a 250 W hardware prototype, experimental results validate the converter’s capability to deliver elevated voltage earning and efficiency under load conditions, outperforming comparable designs in the literature. The converter’s efficiency, with peak values reaching 97%, demonstrates the prospect of this design to enhance the performance and cost-effectiveness of renewable energy applications. This QBC topology, with its enhanced power density and soft-switching capability, sets a new benchmark for high-performance, non-isolated converters in solar power technology.

## Data Availability

The datasets used and/or analysed during the current study are available from the corresponding author upon reasonable request.

## List of symbols

QBC: Quadratic boost converter
$$\:{L}_{1,\:}\:{L}_{2\:,\:}{L}_{1,\:}$$: Inductors
VCR: Voltage Conversion Ratio
CIN: Coupled Inductor
$$\:D$$: Duty cycle
$$\:{D}_{1},\:{D}_{2},{D}_{3},\:{D}_{o}$$: Diodes
$$\:{V}_{in}$$: Input voltage
ZVS: Zero voltage switching
$$\:{C}_{1,\:}\:{C}_{2\:,\:}{C}_{1,\:}\:{C}_{O\:}$$: Capacitors
VGN: Voltage Gain
VSS: Voltage Stress
ZCS: Zero current switching$\:{S}_{1,\:}\:{S}_{2}$ Switches
$\:{C}_{S1,\:}{C}_{S2\:}$: Body capacitance of the switches
$\:\varDelta\:{I}_{Lm}$: Peak ripple current of the magnetizing inductor
$\:{\:Z}_{o}\:$: Impedance of the resonant network
ZVT: Zero voltage transition
$$\:{V}_{O}$$: Output voltage
$$\:{L}_{k\:}$$: Magnetizing inductor
$$\:n$$: Turn ratio
$\:\varDelta\:{i}_{L1}$: Peak inductor current ripple
$\:{V}_{G1,\:}\:{V}_{G2}$: PWM pulse to switches
$$\:{f}_{s\:}$$: Switching frequency
$$\:{L}_{LK\:}$$: Leakage inductor
R: Load resistance
CCM: Continuous conduction mode
ZCT: Zero current transition
$$\:{S}_{1,\:}\:{S}_{2}$$: Switches
